# Effects of Spatial Patch Arrangement and Scale of Covarying Resources on Growth and Intraspecific Competition of a Clonal Plant

**DOI:** 10.3389/fpls.2016.00753

**Published:** 2016-06-06

**Authors:** Yong-Jian Wang, Xue-Ping Shi, Xue-Feng Meng, Xiao-Jing Wu, Fang-Li Luo, Fei-Hai Yu

**Affiliations:** ^1^School of Nature Conservation, Beijing Forestry UniversityBeijing, China; ^2^College of Horticulture and Forestry Sciences, Huazhong Agricultural UniversityWuhan, China

**Keywords:** clonal growth, *Iris japonica*, intraspecific interactions, reciprocal patchiness, pararell patchiness, patch scale

## Abstract

Spatial heterogeneity in two co-variable resources such as light and water availability is common and can affect the growth of clonal plants. Several studies have tested effects of spatial heterogeneity in the supply of a single resource on competitive interactions of plants, but none has examined those of heterogeneous distribution of two co-variable resources. In a greenhouse experiment, we grew one (without intraspecific competition) or nine isolated ramets (with competition) of a rhizomatous herb *Iris japonica* under a homogeneous environment and four heterogeneous environments differing in patch arrangement (reciprocal and parallel patchiness of light and soil water) and patch scale (large and small patches of light and water). Intraspecific competition significantly decreased the growth of *I. japonica*, but at the whole container level there were no significant interaction effects of competition by spatial heterogeneity or significant effect of heterogeneity on competitive intensity. Irrespective of competition, the growth of *I. japonica* in the high and the low water patches did not differ significantly in the homogeneous treatments, but it was significantly larger in the high than in the low water patches in the heterogeneous treatments with large patches. For the heterogeneous treatments with small patches, the growth of *I. japonica* was significantly larger in the high than in the low water patches in the presence of competition, but such an effect was not significant in the absence of competition. Furthermore, patch arrangement and patch scale significantly affected competitive intensity at the patch level. Therefore, spatial heterogeneity in light and water supply can alter intraspecific competition at the patch level and such effects depend on patch arrangement and patch scale.

## Introduction

Spatial heterogeneity in supplies of essential resources (light, water, and soil nutrients) commonly occurs in nature, and different ramets of clonal plants interconnected by, e.g., rhizomes, stolons, and horizontal growing roots are often located in contrasting levels of resource availability (Hutchings and Wijesinghe, 1997; Hutchings and John, 2004; Liu et al., 2006, 2008; Bartels and Chen, 2010). Clonal plants can exhibit foraging responses, i.e., placing more resource-absorbing organs (e.g., leaves, roots, or ramets) in high-quality patches than in low-quality ones, to efficiently utilize heterogeneously distributed resources of light and water (Hutchings and de Kroon, 1994; Hodge, 2004; Hutchings and John, 2004; de Kroon et al., 2005; Guo et al., 2011; Peng et al., 2013). Furthermore, ramets growing in high-quality patches can transport carbohydrates, water and minimal nutrients to those in low-quality ones by physiological integration via rhizomes, stolons, or roots (Alpert and Stuefer, 1997; Price and Marshall, 1999; He et al., 2010, 2011). Such a cooperative system can buffer effects of spatial heterogeneity (Roiloa et al., 2007) and enhance performance of the whole plant (Roiloa and Retuerto, 2007; Hutchings and Wijesinghe, 2008; Song et al., 2013; Zhang and Zhang, 2013; Dong et al., 2015). Spatial heterogeneity in resource supply may also affect plant–plant interactions (Fransen et al., 2001; Day et al., 2003; Moore and Franklin, 2012; Wang et al., 2012; Li H.L. et al., 2014; Dong et al., 2015). For instance, light heterogeneity increased intraspecific competition in *Duchesnea indica* (Wang et al., 2012), and soil nutrient heterogeneity increased intraspecific competition in *Briza media* and interspecific competition between *Festuca ovina* and *B. media* (Day et al., 2003). So far, however, studies testing effects of resource heterogeneity on plant–plant interactions considered spatial heterogeneity in the supply of only one single resource (light or soil nutrients), and little study has examined effects of spatial heterogeneity in two co-variable resources such as light and soil water on intraspecific competition of plants.

In nature, light and soil water commonly co-vary (Alpert and Mooney, 1996). In some habitats such as forest edges, grasslands and shrublands, high light intensity in open patches without vegetation is commonly accompanied with low soil water availability due to high evaporation, and low light intensity underneath dense vegetation is associated with high soil water availability due to low evaporation (Alpert and Mooney, 1996; Griffith, 2010; Li Q.Y. et al., 2014). In such environments with reciprocal patchiness of light and soil water, neither patches alone are ideal for plants growing in them (He et al., 2011; Zhang and Zhang, 2013; Li Q.Y. et al., 2014). In some other habitats such as wetlands or forest gaps opened by disturbance or mortality and dunes with dense shrubs, high light intensity may be associated with high soil water availability and low light intensity with low soil water availability (Prati and Schmid, 2000; Dyer et al., 2010). In such environments with parallel patchiness of light and soil water, patches with high light and high soil water are ideal for plants, whereas patches with low light and low water may not (He et al., 2011; Zhang and Zhang, 2013). Previous studies have shown that reciprocal and parallel patchiness may differently affect the growth of clonal plants (Alpert and Mooney, 1996; Prati and Schmid, 2000; Griffith, 2010; He et al., 2011; Zhang and Zhang, 2013; Li Q.Y. et al., 2014). However, no study has tested whether such patch arrangement (i.e., reciprocal vs. parallel patchiness) affects intraspecific competition of clonal plants. Furthermore, responses of intraspecific competition to resource heterogeneity may also vary with the scale of the patchiness, because foraging ability and thus the growth of plants depends on patch scale of heterogeneity (van der Waal et al., 2011; Wang et al., 2012; Peng et al., 2013; Dong et al., 2015).

To test effects of patch arrangement (reciprocal vs. parallel patchiness) and patch scale on intraspecific competition, we conducted a greenhouse experiment with a rhizomatous, clonal plant *Iris japonica.* We grew one (without intraspecific competition) or nine isolated ramets (with competition) of *I. japonica* under a homogeneous environment and four heterogeneous environments differing in patch arrangement (reciprocal vs. parallel patchiness of light and soil water) and patch scale (large vs. small patches of light and water). Specifically, we addressed the following questions: (1) Does spatial heterogeneity in light and soil water affect intraspecific competition of *I. japonica*? (2) Do reciprocal and parallel patch arrangements have different effects on intraspecific competition of *I. japonica*? (3) Does spatial scale of heterogeneity matter?

## Materials and Methods

### Plant Material

*Iris japonica* Thunb. (Iridaceae) is a perennial clonal herb and widely distributed in forest understories, forest gaps, forest edges, and moist grasslands in Asia (Wang et al., 2013; Li Q.Y. et al., 2014). This species produce long slender rhizomes along which rooted ramets are formed. In the field, most rhizomes are distributed in the top soil of less than 5 cm deep. Inter-ramet distance (spacer length) is 5–15 cm (Wang et al., 2013). Rhizomes that connected ramets of the same genet can break due to disturbance or senescence so that genets become fragmented in the field. The blossoming time is from March to April, and viable seeds are produced from May to June. Clonal growth is the main means for the maintenance and spread of the populations (Wang et al., 2013).

In early January 2014, more than 1000 ramets of *I. japonica* were collected from five locations in an evergreen broad-leaved forest on Shizi Mountain in Hubei Province, China (N 30°28′-30°30′; E 114°20′-114°23′). Adjacent locations were at least 100 m apart so that ramets from different locations were likely to belong to different genotypes. Plants from different locations were mixed and propagated vegetatively in a greenhouse of Huazhong Agricultural University in Hubei Province, China. After 2 weeks of cultivation, we selected 424 similar-sized ramets of *I. japonica*, each with a node, three leaves and some roots. Of them, 24 ramets were randomly selected for measuring initial dry mass (0.389 ± 0.039 g, mean ± SE), and the other 400 were used for the experiment described below.

### Experimental Design

**FIGURE 1:**
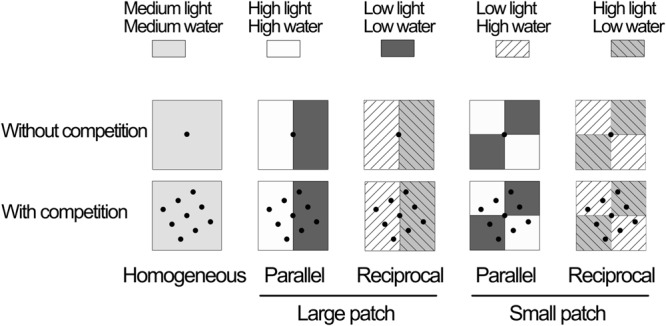
**Experimental design.** The experiment had two competition treatments (without vs. with competition by growing one or nine ramets of *Iris japonica* in a container) crossed with five heterogeneity treatments, i.e., (i) homogeneous (all patches received moderate light and moderate water), (ii) parallel large patch (the container was divided into two large patches; one received high light and high water, and the other low light and low water), (iii) reciprocal large patch (the whole container was divided into two large patches; one patch received high light and low water, and the other low light and high water), (iv) parallel small patch (the container was divided into four small patches; two received high light and high water, and the other low light and low water) and (V) reciprocal small patch (the container was divided into four small patches; two received high light and low water, and the other low light and high water). The light and water content received by the plants in the heterogeneous treatments was the same as that in the homogeneous treatment.

The experiment was a factorial design with two levels of intraspecific competition (without and with intraspecific competition) and five levels of heterogeneity (homogeneous, reciprocal large patch, reciprocal small patch, parallel large patch, and parallel small patch), making a total of 10 treatments (**Figure 1**). In the treatments without competition, one ramet of *I. japonica* was planted in the center of a plastic container (50 cm long × 50 cm wide × 30 cm high) with sealed bottom, and in the treatments with competition, nine ramets were planted (**Figure 1**). In the reciprocal large-patch treatments, each container was divided into two large patches (each measuring 50 cm × 25 cm), one of which was subjected to high light and low water and the other to low light and high water. In the reciprocal small-patch treatments, each container was divided into four small patches (each measuring 25 cm × 25 cm), two of which were subjected to high light and low water and the other two to low light and high water. In the parallel large-patch treatments, each container was divided into two large patches, one of which was subjected to high light and high water and the other to low light and low water. In the parallel small-patch treatments, each container was divided into four small patches, two of which were subjected to high light and high water and the other two to low light and low water. In the homogeneous treatments, each container was subjected to medium light and medium water. There were eight replicates in each treatment.

Each container was filled with a mix of sand and yellow–brown soil (1:1 v/v) homogeneously mixed with 20 g slow release fertilizer (Osmocote, N–P–K: 15–9–12, lasting for 5–6 months). Ramets were transplated to the containers on 14 February 2014 and allowed to recover and establish by supplying with sufficient water for 1 week. Then the soils were allowed to dry for 5 days without adding any water. High light was 100% of natural light in the greenhouse, without covering the patches with a shading net; medium and low light were 55 and 10% of natural light in the greenhouse, respectively, realized by covering the patches with black, neutral shading nets of 55 and 10% transmittances. During the experiment, we added 440 mL water to each container every one to four days depending on the weather conditions and thus how fast the soil dried. In the homogeneous treatments, we spayed 440 mL water evenly to the soil in each container to creat medium water availability. In the heterogeneous treatments with large patches, we supplied 400 mL water to the large patch of high water availability and 40 mL water to the large patch of low water availability in each container. In the heterogeneous treatments with small patches, we added 200 mL water to each of the two small patches of high water availability and 20 mL to each of the two small patches of low water availability in each container.

The bottom of the container was sealed so that there was no water leakage. We built physical barriers between patches inside each container. The barriers were 25 cm high and sealed to the containers (30 cm high) to prevent horizontal flow of water in the soil more than 5 cm deep between patches. Because in the top 5-cm-deep soil, there were no barriers the ramet in the central position in the container could be planted in the soil on the barrier (to be revised further). During watering, we also sprayed water slowly and carefully into each patch to avoid massive horizontal flow of water in the top soil of 5 cm deep. Because rhizomes of *I. japonica* are distributed within the top, 5-cm-deep soil (Wang et al., 2013), the physical barriers could not prevent rhizomes to grow across patches. Soil water content was monitored everyday in four replications during the experiment by a Soil Moisture Meter (TZS-II, HEB Biotechnology Co., Xi’an, China). Soil water content was about 32–37% in the high water patches, 20–25% in the medium water patches and 8–13% in the low water patches.

The experiment was conducted in the greenhouse at Huazhong Agricultural University. During the experiment, the mean temperature and mean relative humidity in the greenhouse were 25.1°C and 72.2%, respectively (measured by Amprobe TR300, Amprobe, Everett, WA, USA). Light intensity in the greenhouse was 85% of that outside. The experiment was started on 26 February 2014 and ended on 1 July 2014, lasting for 125 days.

### Measurements

At the end of the experiment, parent (original) ramets and offspring ramets were harvested separately. For the reciprocal patch treatments, we harvested offspring ramet located in the patches of high water and low light and patches of low water and high light separately. Similarly, for the parallel patch treatments, we harvested offspring ramets in the patches of high water and high light and patches of low water and low light separately. In each container, we pooled offspring ramets located in the same type of patches into one sample. For the homogeneous treatment, offspring ramets were harvested in a similar fashion, i.e., offspring ramets located in the imagined high and low water patches were harvested separately and those in the same type of imaged patches were pooled into one sample. The plants were then separated into leaves, stem, rhizomes, and roots, dried at 80°C for 48 h and weighed. Biomass in a container (at the container level) was the sum of biomass of the parent ramets, offspring ramets located in the high water patches and offspring ramets in the low water patches in that container. Similarly, we obtained number of ramets and rhizome length at the container level.

### Data Analysis

The growth measures could not be compared directly because number of initial ramets of *I. japonica* differed between the two competition treatments (one vs. nine for the treatments without vs. with competition). Thus, we calculated biomass, number of ramets and rhizome length on the basis of per initial ramet in each container and also in each type of the patches, and these data were used for further analyses.

We used two-way ANOVAs to test effects of intraspecific competition (with and without competition) and spatial heterogeneity (homogeneous, reciprocal large and small patch, and parallel large and small patch) on the growth of *I. japonica* at the container level. If a significant effect of spatial heterogeneity was detected, then Tukey HSD tests to conducted to compare the means among the five heterogeneity treatments. The aim of these analyses was to examine whether there was an overall impact of spatial heterogeneity (homogeneous treatment vs. heterogeneous treatments of different types), as well as its interaction with intraspecific competition, so that the homogeneous treatments could be included. We further used three-way ANOVAs to examine effects of intraspecific competition, patch arrangement (reciprocal vs. parallel) and patch scale (small vs. large) on the growth at the container level, and in these analyses the homogeneous treatments were excluded. The aim of these analyses was to test the effect of patch scale and patch arrangement (and their interactions with competition), and the homogeneous treatments could not be included because they did not belong to either of the two patch sizes or patch arrangements. At the patch level, we employed three-way ANOVAs with repeated measures to test effects of intraspecific competition, spatial heterogeneity and patch type (high vs. low water patches) within a container on the growth of offspring ramets of *I. japonica* (Wang et al., 2012). If a significant effect of spatial heterogeneity was detected, then Tukey HSD tests to conducted to compare the means among the five treatments. We further used four-way ANOVAs with repeated measures to test effects of intraspecific competition, patch arrangement, patch scale and patch type within a container on the growth of offspring ramets at the patch level (Wang et al., 2012; Dong et al., 2015), and in these analyses the homogeneous treatments were excluded. Patch type within a container was used as a repeated variable because the two types of patches in a container were not independent (Potvin et al., 1990; Zar, 1999, p. 255).

To measure the intraspecific competitive intensity, we calculated the log response ratio (LnRR) of biomass as LnRR = ln(*B*_o_/*B*_w_), where *B*_o_ is mean biomass of a treatment without competition across the eight replicates, and *B*_w_ is biomass of the treatment with competition in each replicate. Values of LnRR are symmetrical around zero (Hedges et al., 1999; Armas et al., 2004). Positive values indicate competition, negative values indicate facilitation and zero indicates neutral. At the container level, we used one-way ANOVA to examine the effect of spatial heterogeneity on LnRR. If a significant effect was detected, we further used two-way ANOVA to test the effects of patch arrangement and patch scale on LnRR. At the patch level, we used two-way ANOVA to examine the effect of spatial heterogeneity and patch type on LnRR. If a significant effect of spatial heterogeneity was detected, we further used three-way ANOVA to test the effects of patch arrangement, patch scale and patch type on LnRR. Patch type was treated as a repeated variable. All analyses were conducted using SPSS 13.0 (SPSS, Chicago, IL, USA).

## Results

### Effects of Spatial Heterogeneity and Intraspecific Competition at the Container Level

**FIGURE 2:**
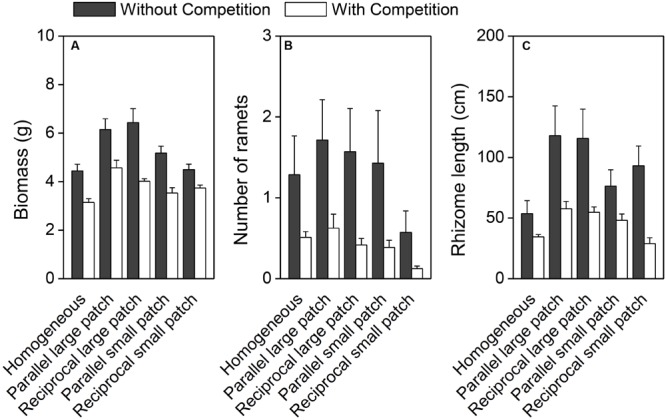
**Biomass **(A)**, number of ramets **(B)**, and rhizome length **(C)** of *I. Japonica* at the whole container level under the ten treatments.** Error bars show +SE.

Spatial heterogeneity in light and water significantly affected biomass and rhizome length of *I. japonica* at the container level (**Table 1**). Irrespective of competition, biomass and rhizome length were the highest in the heterogeneous treatments with large patches, smallest in the homogeneous treatments, and intermediate in the heterogeneous treatments with small patches (**Figures 2A,C**; **Appendices 1A,C**; **Tables 1** and **2**). However, none of the three growth measures differ significantly between parallel and reciprocal patch arrangements (**Figure 2**; **Appendices 1A–C**; **Table 2**).

**Table 1:** ANOVAs for effects of spatial heterogeneity (homogeneous vs. parallel large patch vs. reciprocal large patch vs. parallel small patch vs. reciprocal small patch) and intraspecific competition (without vs. with competition) on the growth of *Iris japonica* at the whole container level.

Effect	df	Biomass	Number of ramets	Rhizome length
Heterogeneity (H)	4, 80	**5.365^∗∗^**	1.277	**2.812^∗^**
Competition (C)	1, 80	**55.727^∗∗∗^**	**10.992^∗∗^**	**31.977^∗∗∗^**
H × C	4, 80	1.107	0.234	1.176

*Significance levels: ^∗∗∗^*P* < 0.001, ^∗∗^*P* < 0.01, ^∗^*P* < 0.05.*

**Table 2:** ANOVAs for effects of patch arrangement (parallel vs. reciprocal), patch scale (large vs. small) and intraspecific competition (without vs. with competition) on the growth of *I. japonica* at the whole container level.

Effect	df	Biomass	Number of ramets	Rhizome length
Patch arrangement (*P*_a_)	1, 64	1.373	0.894	0.032
Patch scale (*P*_s_)	1, 64	**8.927^∗∗^**	**6.502^∗^**	**4.253^∗^**
Competition (C)	1, 64	**45.823^∗∗∗^**	**8.716^∗∗^**	**28.749^∗∗∗^**
*P*_a_ ×*P*_s_	1, 64	0.171	0.028	0.001
*P*_a_ × C	1, 64	0.822	0.024	0.857
*P*_s_ × C	1, 64	0.940	1.743	0.053
*P*_a_ ×*P*_s_ × C	1, 64	0.260	0.002	0.688

*Significance levels: ^∗∗∗^*P* < 0.001, ^∗∗^*P* < 0.01, ^∗^*P* < 0.05.*

**FIGURE 3:**
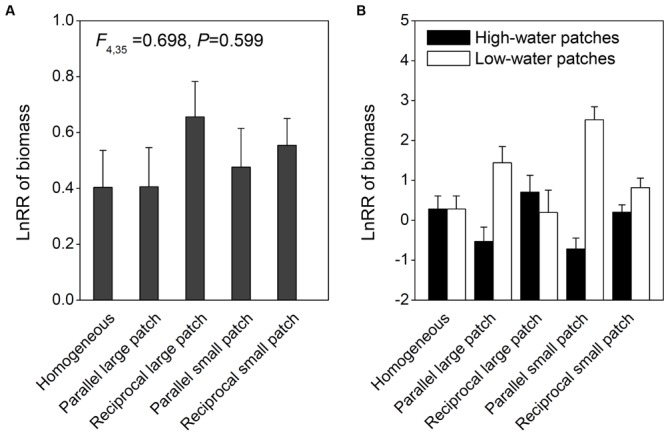
**Completive intensity as measured by log response ratio (LnRR) of biomass of *I. japonica* at the whole container level **(A)** and in the high and low water patches at the patch level **(B)** in the five treatments.** Error bars show +SE.

Intraspecific competition significantly decreased biomass, number of ramets and rhizome length of *I. japonica* at the container level (**Figure 2**; **Tables 1** and **2**). However, there were no significant interaction effects of competition by spatial heterogeneity (**Tables 1** and **2**; **Figure 2**), and no significant effect of spatial heterogeneity on the log response ratio of biomass (LnRR; **Figure 3A**, *F*_4,35_ = 0.698, *P* = 0.599), suggesting that spatial heterogeneity in light and water, irrespective of its patch arrangement or scale, did not alter intraspecific competitive intensity of *I. japonica* at the container scale.

### Effects of Spatial Heterogeneity and Intraspecific Competition at the Patch Level

**FIGURE 4:**
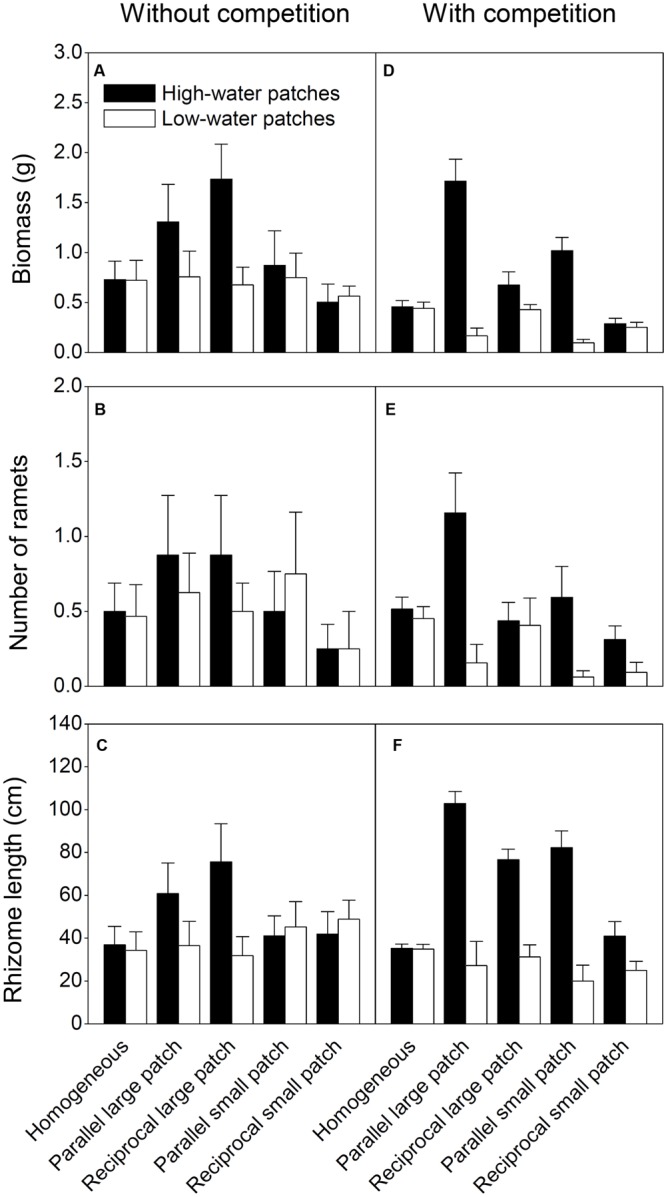
**Biomass **(A,D)**, number of ramets **(B,E)** and rhizome length of **(C,F)***I. japonica* in the high and low water patches under the ten treatments.** Error bars show +SE.

There were significant interaction effects of patch type × spatial heterogeneity (**Table 3**), patch type × spatial heterogeneity × competition (**Table 3**), patch type × patch arrangement (**Table 4**), patch type × patch arrangement × patch scale (**Table 4**), and patch type × patch arrangement × competition (**Table 4**) on the growth measures. Irrespective of competition, biomass, number of ramets and rhizome length were significantly larger in the high than in the low water patches in the heterogeneous treatments with large patches, but was statistically the same in the high and low water patches in the homogeneous treatments (**Figure 4**; **Tables 3** and **4**). For the heterogeneous treatments with small patches, the growth of *I. japonica* was not significantly affected by patch type in the absence of competition, but significantly larger in the high than in the low water patches in the presence of competition (**Figure 4**; **Appendices 1D–F**; **Tables 3** and **4**).

**Table 3:** ANOVAs for effects of heterogeneity (homogeneous vs. parallel large patch vs. reciprocal large patch vs. parallel small patch vs. reciprocal small patch), intraspecific competition (without vs. with competition) and patch type (high vs. low water patches) on the growth of *I. japonica* at the patch level.

Effect	df	Biomass	Number of ramets	Rhizome length
Heterogeneity (H)	4, 80	**5.401^∗∗∗^**	1.812	**6.716^∗∗∗^**
Competition (C)	1, 80	0.001	1.900	0.011
Patch type (*P*_t_)	1, 80	**175.642^∗∗∗^**	1.658	0.445
H × C	4, 80	**6.936^∗∗∗^**	0.182	1.683
H ×*P*_t_	4, 80	**45.715^∗∗∗^**	**2.557^∗^**	**13.536^∗∗∗^**
C ×*P*_t_	1, 80	**63.164^∗∗∗^**	2.452	1.467
H × C ×*P*_t_	4, 80	**10.875^∗∗∗^**	1.559	**4.990^∗∗^**

*Significance levels: ^∗∗∗^*P* < 0.001, ^∗∗^*P* < 0.01, ^∗^*P* < 0.05. Patch type was treated as a repeated variable.*

**Table 4:** ANOVAs for effects of patch arrangement (parallel vs. reciprocal), patch scale (large vs. small), intraspecific competition (without vs. with competition) and patch type (high vs. low water patches) on the growth of *I. japonica* at the patch level.

Effect	df	Biomass	Number of ramets	Rhizome length
Patch arrangement (*P*_a_)	1, 64	**4.096^∗^**	2.220	0.516
Patch scale (*P*_s_)	1, 64	**11.594^∗∗∗^**	**4.303^∗^**	**3.879^∗^**
Competition (C)	1, 64	**4.517^∗^**	1.728	0.085
Patch type (*P*_t_)	1, 64	**233.102^∗∗∗^**	1.104	**3.745^∗^**
*P*_a_ ×*P*_s_	1, 64	0.007	0.144	0.029
*P*_a_ × C	1, 64	3.230	0.021	3.359
*P*_a_ ×*P*_t_	1, 64	**58.490^∗∗^**	**6.249^∗^**	**27.417^∗∗∗^**
*P*_s_ × C	1, 64	0.001	0.001	1.411
*P*_s_ ×*P*_t_	1, 64	0.519	0.820	0.658
C ×*P*_t_	1, 64	**40.372^∗∗∗^**	**3.987^∗^**	2.368
*P*_a_ ×*P*_s_ × C	1, 64	0.506	0.622	0.197
*P*_a_ ×*P*_s_ ×*P*_t_	1, 64	**11.486^∗∗∗^**	1.797	**7.337^∗∗^**
*P*_a_ × C ×*P*_t_	1, 64	**36.669^∗∗∗^**	2.658	**14.505^∗∗∗^**
*P*_s_ × C ×*P*_t_	1, 64	0.558	0.379	0.009
*P*_a_ ×*P*_s_ × C ×*P*_t_	1, 64	0.241	0.474	0.592

*Significance levels: ^∗∗∗^*P* < 0.001, ^∗∗^*P* < 0.01, ^∗^*P* < 0.05. Patch type was treated as a repeated variable.*

There were significant interaction effects of patch type × spatial heterogeneity (*F*_4,35_ = 7.815, *P* < 0.001), patch type × patch arrangement (*F*_1,28_ = 17.634, *P* < 0.001) and patch type × patch scale (*F*_1,28_ = 6.705, *P* = 0.036) on LnRR at the patch level (**Figure 3B**). LnRR was significantly larger in the low than in the high water patches in the heterogeneous treatments with the parallel arrangement, but was statistically the same in the high and low water patches in the homogeneous treatments and the heterogeneous treatments with the reciprocal arrangement (**Figure 3B**). LnRR was significantly larger in the low than in the high water patches in the heterogeneous treatments with small patches, but was not significantly affected by patch type in the reciprocal arrangement with large patches (**Figure 3B**). Compared to the homogeneous treatments and the reciprocal arrangement treatments, the parallel arrangement treatments greatly decreased LnRR in the high water patches, but increased that in the low water patches (**Figure 3B**). These results suggest that patch type, patch arrangement and patch scale can alter intraspecific competitive intensity of *I. japonica* at the patch scale.

## Discussion

While many studies have tested effects of environmental heterogeneity in the supply of a single resource (e.g., light or soil nutrients) on intraspecific and/or interspecific interactions of plants (Fransen et al., 2001; Day et al., 2003; Moore and Franklin, 2012; Wang et al., 2012; Li H.L. et al., 2014; Dong et al., 2015), none has examined those of heterogeneous distribution of two co-variable resources. Our results clearly showed that spatial heterogeneity in light and water availability could alter intraspecific competition at the patch level and that such effects depended on spatial patch arrangement and patch scale.

Spatial heterogeneity in light and water availability, irrespective of its patch arrangement or patch scale, did not significantly alter intraspecific competition intensity of *I. japonica* at the container scale. Previous studies have also showed that soil nutrient heterogeneity did not affect intraspecific competition in *Hydrocotyle vulgaris* (Dong et al., 2015), *Alternanthera philoxeroides* (Zhou et al., 2012) or *F. ovina* (Day et al., 2003) at the container level. It has been suggested that a significant effect of light and soil heterogeneity on competition may be caused by the differences between plants in their ability to concentrate ramets and/or roots where resource levels are high (Fransen et al., 2001; Zhou et al., 2012). In this study, *I. japonica* showed the relatively high and low ability to concentrate ramets and rhizome mass in high and low water patches, respectively. Consequently, a significant integrative effect of resource heterogeneity of light and water on the intraspecific interactions was not observed at the container level.

In the heterogeneous treatment with parallel patchiness, resource heterogeneity of light and water significantly decreased intraspecific competition intensity of *I. japonica* in the high water patches and increased that in the low water patches, but such effects were absent in the heterogeneous treatments with reciprocal patchiness. Thus, at the patch level, patchy distribution of light and water could alter intraspecific competition and patch arrangement mattered. Previous studies indicated that preferential ramet and root placements in resource-rich patches might greatly improve the efficiency and amount of resource capture and further increase their local growth (Roiloa and Retuerto, 2007; Wang et al., 2012). The efficiency of resource capture by these ramets in resource-rich patches can also benefit the growth of the whole plant by resource translocation from connected ramets in competition-free conditions (He et al., 2011; Zhou et al., 2012; Zhang and Zhang, 2013). In the presence of competition, plants prefer to concentrate more new ramets or new rhizomes in the high water and high light patches to promote the success of growth rather than in the low water and low light patches. These may result from the fact that resources were sufficient in high water and high light conditions and insufficient in low water and low light conditions for the competitive growth of *I. japonica*. Clonal integration might reduce the intensity of competition between ramets in the resource-rich patches by allowing internal transportation of resources from the ramets in the resource-rich patches to the connected ramets in the resource-poor patches, which can also be beneficial to plant growth in resource-rich patches (Novoplansky, 2009; Dong et al., 2015). In reciprocal patchiness, *I. japonica* might develop division of labor of plants between high water with low light and low water with high light conditions, which shared the risk of intense competition in both high and low water patches (Stuefer et al., 1996; Wang et al., 2011). That might be also a strategy for selecting advantageous patches and balancing the benefit between resource-poor and resource-rich patches in competition conditions.

Impacts of patchy distribution of two co-variable resources on intraspecific competition intensity of *I. japonica* also depended on patch scale. Patch scale had substantial effects on performance of clonal plants (Wijesinghe and Hutchings, 1997, 1999), and resource heterogeneity that affects plant performance at one scale may not do so at other scales. For instance, *Glechoma hederacea* clones growing in heterogeneous conditions with large patches produced greater biomass than those growing in heterogeneous conditions with small patches (Wijesinghe and Hutchings, 1997, 1999). However, spatial heterogeneity in light intensity increased intraspecific competition intensity of *D. indica* at both large and small patch scales (Wang et al., 2012). Impacts of patch scale on plant performance and interaction may be related to inter-ramet distance and also the size of root and shoot systems of ramets. If the patch size is too small or too large, then there will be no impact of heterogeneity (Zhou et al., 2012; Dong et al., 2015). In our study, enough space with sufficient resources in large patches with high water availability was benefit to the competitive growth of *I. japonica*. However, in small patches with high water availability, the rhizome growth and placement of new ramets of *I. japonica* was restricted, and nearly all space was overloaded. Thus the resource-rich patches might not always maintain equal suitability and gradually decline to the same level of suitability as the resource-poor patches. Therefore, in our study, plants in heterogeneous treatments with large patches produced more new ramets or new rhizome in the high water than low water patches in competitive conditions.

Our results also indicate that *I. japonica* exhibited foraging responses in the heterogeneous environment of both reciprocal and parallel patchiness, especially in competitive conditions. The possible reason can be the existence of a negative correlation between the space of plant growth and foraging precision (Wijesinghe et al., 2001; Cahill et al., 2010). If *I. japonica* grows alone, sufficient space may favor the fast growth of plants and decrease its foraging precision, which might lead to ignoring the heterogeneous resource distribution in the parallel patchiness (Rajaniemi and Reynolds, 2004; Mommer et al., 2012; Dong et al., 2015). In reciprocal patchiness, *I. japonica* might develop division of labor of plants between high water with low light and low water with high light conditions, which promoted the high potential benefits to enhance resource capture of clonal plants and thereby to increase their performance in heterogeneous habitats (Stuefer et al., 1996; Roiloa et al., 2007; Wang et al., 2011; Zhang and Zhang, 2013). Meanwhile, in the presence of intraspecific competition, limited space for the growth may enable *I. japonica* to show a higher foraging precision in response to resources (Cahill et al., 2010; Dong et al., 2015), especially in parallel patchiness with extremely rich and poor patches.

## Conclusions

We conclude that environmental heterogeneity in the supply of two co-variable resources can affect intraspecific interactions of plants at some circumstances. Our results also suggest that competitive responses to spatial heterogeneity in resource availability may necessarily be adaptive and depend on resource combination and patch scale. Therefore, spatial heterogeneity in light and water availability may be of great importance in regulating population structure and dynamics of clonal plants (Hutchings et al., 2003; He et al., 2011; Wang et al., 2012; Zhang and Zhang, 2013).

## Author Contributions

Y-JW, X-PS, and F-HY designed the experiment. X-JW and X-FM performed the experiment. Y-JW wrote the first draft of the manuscript. Y-JW, F-LL, and F-HY did the statistical analysis. Y-JW and F-HY contributed substantially to the revisions.

## Conflict of Interest Statement

The authors declare that the research was conducted in the absence of any commercial or financial relationships that could be construed as a potential conflict of interest.

## Supplementary Material

DATA SHEET 1 **Biomass **(A,D)**, number of ramets **(B,E)** and rhizome length **(C,F)** of *Iris japonica* at the whole container level (i) and at the patch level (ii) under the five heterogeneity treatments.** Error bars show +SE. Letters show the differences between the treatments (Tukey HSD tests, *P* = 0.05).
