# Supplementary figures and images for: Effects of Spatial Patch Arrangement and Scale of Covarying Resources on Growth and Intraspecific Competition of a Clonal Plant
Source: Front Plant Sci. 2016 Jun 6;7:753. doi: 10.3389/fpls.2016.00753 (PMC4891989; doi:10.3389/fpls.2016.00753)

(i)

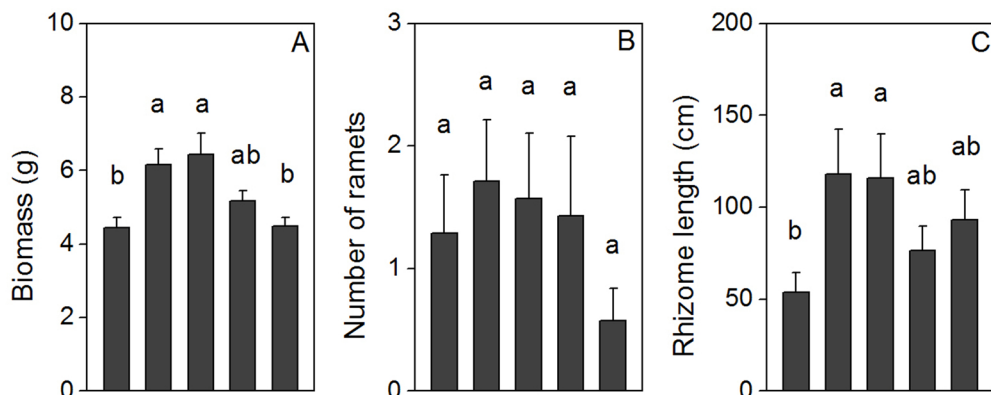

(ii)

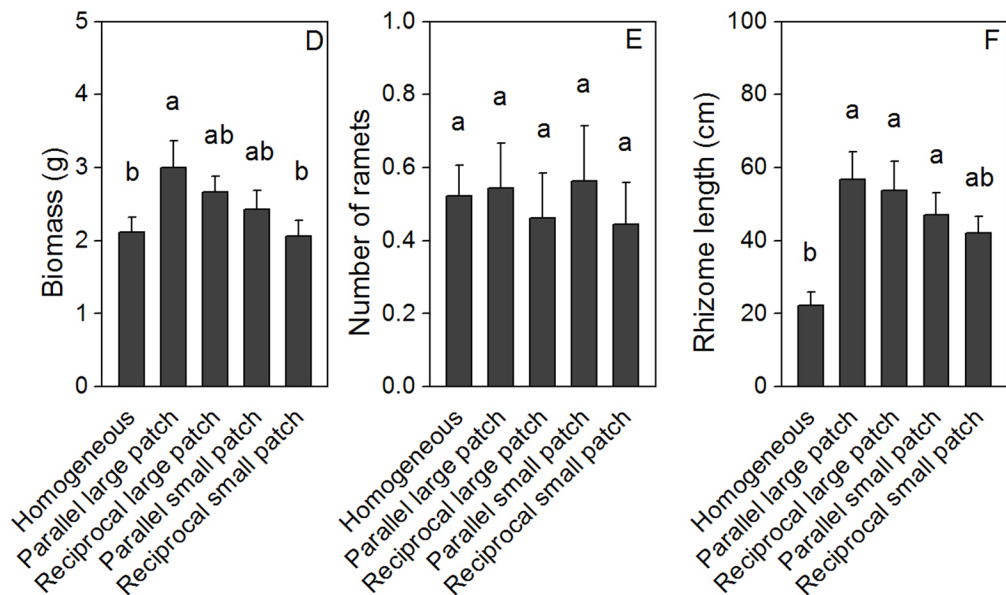

Supplement: DATA SHEET 1 — Biomass (A,D), number of ramets (B,E) and rhizome length (C,F) of Iris japonica at the whole container level (i) and at the patch level (ii) under the five heterogeneity treatments. Error bars show +SE. Letters show the differences between the treatments (Tukey HSD tests, P = 0.05). [file Data_Sheet_1.PDF]
